# Effectiveness and cost effectiveness of palliative care interventions in people with chronic heart failure and their caregivers: a systematic review

**DOI:** 10.1186/s12904-022-01092-2

**Published:** 2022-11-23

**Authors:** Stephanie Hicks, Martin Davidson, Nikolaos Efstathiou, Ping Guo

**Affiliations:** 1 St George’s University Hospitals NHS Foundation Trust, London, UK; 2 Cicely Saunders Institute of Palliative Care, Policy & Rehabilitation, Florence Nightingale Faculty of Nursing, Midwifery & Palliative Care, King’s College London, London, UK; 3 Blackpool Teaching Hospitals NHS Foundation Trust, Blackpool, UK; 4 School of Nursing and Midwifery, Institute of Clinical Sciences, College of Medical and Dental Sciences, University of Birmingham, Birmingham, UK

**Keywords:** Chronic heart failure, Palliative care, Effectiveness, Cost effectiveness, Interventions, Systematic review

## Abstract

**Background:**

Chronic heart failure is a common condition, and its prevalence is expected to rise significantly over the next two decades. Research demonstrates the increasing multidimensional needs of patients and caregivers. However, access to palliative care services for this population has remained poor. This systematic review was to provide an evidence synthesis of the effectiveness and cost-effectiveness of palliative care interventions for people with chronic heart failure and their caregivers.

**Methods:**

Relevant publications were identified via electronic searches of MEDLINE, Embase, PsychInfo, CINAHL, CENTRAL and HMIC from inception to June 2019. Grey literature databases, reference list, and citations of key review articles were also searched. Quality was assessed using the Revised Cochrane Risk of Bias Tool.

**Results:**

Of the 2083 records, 18 studies were identified including 17 having randomised controlled trial (RCT) designs and one mixed methods study with an RCT component. There was significant heterogeneity in study settings, control groups, interventions delivered, and outcome measures used. The most commonly assessed outcome measures were functional status (*n* = 9), psychological symptoms (n = 9), disease-specific quality of life (n = 9), and physical symptom control (*n* = 8). The outcome measures with the greatest evidence for benefit included general and disease-specific quality of life, psychological symptom control, satisfaction with care, physical symptom control, medical utilisation, and caregiver burden. Moreover, the methodological quality of these studies was mixed, with only four having an overall low risk of bias and the remaining studies either demonstrating high risk of bias (*n* = 10) or showing some concerns (*n* = 4) due to small sample sizes and poor retention. Only two studies reported on economic costs. Both found statistically significant results showing the intervention group to be more cost effective than the control group, but the quality of both studies was at high risk of bias.

**Conclusions:**

This review supports the role of palliative care interventions in patients with chronic heart failure and their caregivers across various outcomes, particularly quality of life and psychological wellbeing. Due to the highly heterogeneous nature of palliative care interventions, it is not possible to provide definitive recommendations as to what guise palliative care interventions should take to best support the complex care of this population. Considerable future research, particularly focusing on quality of care after death and the caregiver population, is warranted.

**Supplementary Information:**

The online version contains supplementary material available at 10.1186/s12904-022-01092-2.

## Background

Chronic heart failure (CHF) is a long-term condition [1] which has been estimated to affect at least 26 million people worldwide [2]. Within developed nations, it has an estimated prevalence of between 1 and 2% of adults [3, 4]. The median prevalence of CHF in adults ≥60 years has been reported to be 11.8% [5] and future projections estimate that this will increase by 50% in the next 20 years [6]. As a consequence of its growing prevalence, CHF has been described as a global public health burden. In 2012, it was estimated to account for £22.5 billion of health expenditure globally and total costs are expected to increase by 127% between 2012 and 2030 [7]. Of note, it has been shown that approximately two-thirds of the economic burden from heart failure is secondary to hospitalisation rates, in particular high re-admission rates [8].

One landmark study in 2001 highlighted the 5-year survival following an initial hospitalisation with heart failure was approximately 25%, worse than many common cancer populations [9]. Since then, a number of large international cohort studies assessing prognosis have demonstrated some improvement in these figures, with 5-year survival in the CHF population now approximately 50–60% compared with 85% in the age-matched general population [10, 11]. The most strongly associated risk factor with regards to poor prognosis is advancing age [10, 12]. Other risk factors include hyponatraemia, anaemia, acute kidney injury, chronic kidney disease, low systolic blood pressure, male sex, and presence of co-morbidities [13].

The challenge in prognostication in CHF is arguably related to the unpredictable nature of the illness trajectory. Compared to most cancers, where there is typically a clear point at which patients begin to deteriorate, CHF classically demonstrates a much more complex trajectory [14]. For example, patients with CHF may have multiple episodes of decompensation requiring hospital admission and within each episode be sick enough to die, but may also have the potential to make a recovery back to their premorbid level. There is also an underlying risk of sudden cardiac death, which increases as the left ventricular ejection fraction falls [15]. Therefore, it can be challenging to identify when future deterioration or the beginning of a dying process may occur and to initiate palliative care interventions.

The role of palliative care is to provide much more than simply physical symptom control, with an equal focus on other key holistic principles of care [16]. For instance, consideration of evidence on advance care planning in the CHF population indicates that, despite being able to indicate their preferences regarding life-sustaining treatments, patients with CHF rarely have the opportunity for these discussions [17]. There is evidence that patients and their caregivers have information needs around CHF, its symptoms and disease progression that are not being met [18]. In addition, studies that have assessed the unmet need of caregivers of patients with CHF have highlighted numerous issues that may add to their burden. These include providing and receiving emotional and spiritual support; having difficult conversations; dealing with uncertainty about the future and the unpredictably of the heart failure; communication and care coordination; handling financial issues; knowing what to expect and how to care for patients at the end of life; and timely access to formal and informal social support and services [19].

Care of patients with CHF and their families is complex, and arguably the holistic approach offered by palliative care may be one way to meet these complex needs. Indeed, in other disease groups, such as cancer, palliative care interventions throughout the disease trajectory have increasingly been recognised as valuable by improving quality of life, symptoms and even survival [20–22]. It is hypothesised that integrating a palliative care approach into the management of patients with CHF may improve symptom control and quality of life, lead to decreased hospital admissions, improve mortality and reduce health costs. A joint World Health Organization (WHO) and the Worldwide Palliative Care Alliance report published in 2014 outlining the global unmet need for palliative care estimated that, of all adults dying in need of palliative care, 38.5% died from cardiovascular diseases [23]. Access to palliative care is now recommended by all major cardiovascular societies in the management of advanced CHF [24, 25].

However, current evidence suggests incorporation of palliative care services into heart failure management has been largely inconsistent [24] and there have been barriers to access to palliative care support for individuals with CHF. Only 6.4% of hospitalised patients with heart failure were referred to palliative care services [26], which is significantly lower than referral rates seen in cancer populations [27]. In a UK setting, a survey of palliative care specialists found that 47% of services received fewer than ten referrals for patients with heart failure in a year, with only 3% of the entire palliative care workload being patients with heart failure [28]. Furthermore, when patients are referred, this often occurs late in the disease course [29]. The aim of this systematic review was to provide an evidence synthesis of the effectiveness and cost-effectiveness of palliative care interventions for people with CHF and their caregivers.

## Methods

This systematic review was undertaken following guidance from the Cochrane Handbook for Systematic Reviews of Interventions [30] and is reported in accordance with the Preferred Reporting Items for Systematic reviews and Meta-Analyses (PRISMA) statement [31]. A protocol was developed, and peer reviewed by experienced systematic reviewers.

### Search strategy

The search strategy was developed by the authors (SH and PG) with input from a Library Information Specialist. Relevant medical subject headings and keywords (such as ‘chronic heart failure’ and ‘palliative care’; the full list of search terms is provided in online supplemental data file Appendix 1) were used to search databases. Six electronic databases [MEDLINE, EMBASE, PsychINFO, CINAHL (EBSCO), Cochrane Central Register of Controlled Trials (CENTRAL), and Health Management Information Consortium (HMIC)] were systematically searched from their inception to June 2019. Grey literature searching was performed by hand-searching reference lists of included studies and key review articles. CareSearch grey literature database, an Australian palliative care network with information on conference abstracts, research studies in progress and non-indexed journals, was also searched.

### Inclusion and exclusion criteria

All peer reviewed studies published in English were included if these studies focused on: (1) adults aged 18 or over with a diagnosis of CHF or acutely decompensated CHF graded at the New York Heart Association (NYHA) II-IV as a primary or secondary diagnosis in any care settings or their informal caregivers; (2) two or more clinical or non-clinical palliative care interventions as outlined by the palliative care workshop of the Heart Failure Association of the European Society of Cardiology [32]; (3) any outcome measures; and (4) used experimental designs (e.g. randomised controlled studies, non-randomised controlled studies, before and after studies, interrupted time series studies, or feasibility or pilot studies).

Studies were excluded if they included (1) people with acute heart failure; (2) paid caregivers; (3) interventions that target only one palliative care domain alone which could not reflect the holistic nature of palliative care (e.g., advance care planning only); (4) non-experimental designs such as observational studies, and (5) studies in progress.

### Study selection

Following completion of searches, references were exported to and managed by RefWorks software which enabled identification and removal of duplicates. Titles and abstracts were screened by one reviewer (SH) for relevance, and some were excluded at this stage. Two reviewers (SH & MD) then independently assessed the remaining full text articles as well as articles identified through grey literature searching against eligibility criteria for inclusion. Any disparities were resolved through discussion between the two reviewers. Where consensus could not be reached, advice was sought from the third reviewer (PG).

### Data extraction

A standardised data extraction form (Excel spreadsheet) was specifically designed and used for this review, based on the guidance from the Cochrane Handbook for Systematic Reviews of Interventions [30]. Study characteristics including participants, the intervention and control groups and all outcomes reported in the included studies were extracted by SH, and independently checked by PG for accuracy and detail. Any disparities were resolved by discussion and consensus (with MD). In the event that data were not recorded within the paper or were ambiguous, attempts were made to contact the authors for clarification. However, if this additional information was unable to be obtained, analysis was performed based on data contained within the paper only.

### Risk of bias assessment

Quality assessment was performed using the Revised Cochrane risk-of-bias tool for randomized trials (RoB 2) [33]. It is a domain-based evaluation tool which encompasses five key domains: Bias arising from the randomization process; bias due to deviations from intended interventions; bias due to missing outcome data; bias in measurement of the outcome; and bias in selection of the reported result. In this review, SH completed risk of bias assessment and any queries were discussed with PG. Should any controlled before-and-after studies (CBAs) or interrupted time series (ITS) studies be identified for inclusion, the Cochrane EPOC group guidance on CBAs and ITS studies was planned to be used [30].

### Data synthesis

Descriptive synthesis of the study design, setting, population, intervention, and comparator was conducted. The study outcomes and results were summarised in a narrative way [34]. Meta-analysis was not considered appropriate, given the significant heterogeneity of the intervention delivered and outcome measures used in the studies included in this review.

## Results

**Fig. 1:**
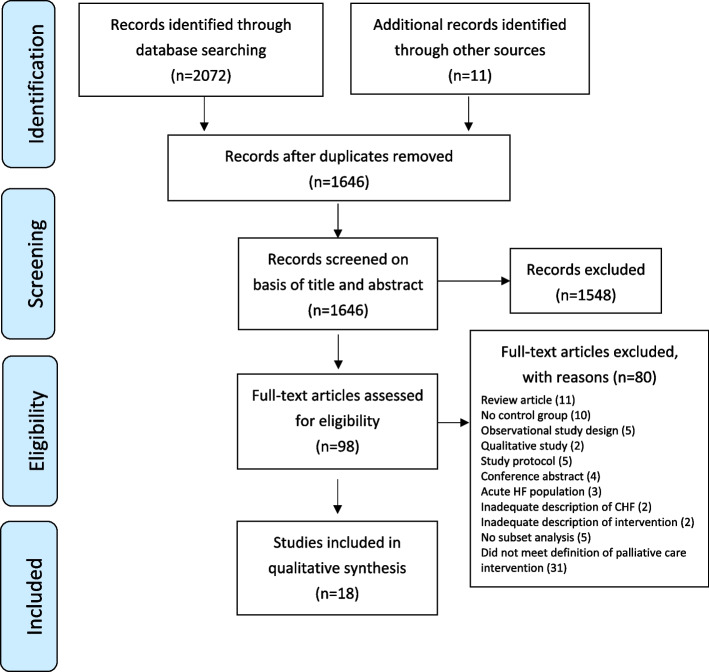
PRISMA flow diagram depicting studies selection process

Initial database searches identified 2072 records and a further 11 studies were identified through grey literature searching. Once duplicates were removed, 1646 records remained, which were screened based on abstracts and titles. A total of 1548 articles were excluded. Subsequently, the full texts of 98 remaining articles were retrieved and assessed against inclusion and exclusion criteria, leaving 18 studies for inclusion in this review (Fig. 1).

### Study characteristics

Studies were conducted across four countries: the USA (*n* = 9) [35–43], Sweden (*n* = 5) [44–48], China (*n* = 3) [49–51], and the UK (*n* = 1) [52]. The majority had a randomised controlled trial (RCT) design (*n* = 17), and one was a pilot mixed methods study with an RCT component [42]. The sample sizes of included studies ranged from 11 [52] to 392 [36]. The setting for care delivery varied across the studies, encompassing the full range of potential sites for palliative care delivery: community (*n* = 4) [35, 49–51], outpatients (*n* = 6) [37, 39, 42, 43, 46, 52], hospital (n = 1) [38], and across mixed settings (*n* = 7) [36, 40, 41, 44, 45, 47, 48]. Table 1 summarises the key study characteristics of each included study.

**Table 1:** Characteristics of included studies (*n* = 18)

First Author, Year, Country	Study Design and Setting	Participants: Sample size (n), Age (years), Sex, Disease characteristics (NYHA Class, LVEF)		Description of Intervention and Control Groups	Outcomes Measured (& Measurement Scale)	Results
		Intervention	Control			
**Agren et al.** [44] **Sweden**	RCT Hospital and community based (outpatient clinic and in patients’ homes)	*N* = 71 dyads (patient & carer) Patients’ Mean Age (SD): 69 (13) Partners’ Mean Age (SD): 67 (12) Sex (patients): M 49 (69.1%), F 22 (30.9%) Sex (partners): M 22 (30.9%), F 49 (69.1%) NYHA Class II 25 (35%) NYHA Class III 39 (55%) NYHA Class IV 7 (10%) No information on LVEF	*N* = 84 dyads (patient & partner) Patients’ Mean Age (SD): 73 (10) Partners’ Mean Age (SD): 70 (10) Sex (patients): M 68 (80.9%), F 16 (19.1%) Sex (partners): M 16 (80.9%), F 68 (19.1%) NYHA Class II 25 (30%) NYHA Class III 43 (51%) NYHA Class IV 16 (19%) No information on LVEF	Intervention HF nurse-led face to face counselling, a computer based CD-ROM program and written teaching materials. 3 sessions in home or clinic: each included education on CHF, development of problem-solving skills in recognising factors contributing to psychological and emotional distress and focused on changing thoughts and behaviours and implementing strategies for self-care. Opportunity for dyads to receive information, raise questions, discuss difficulties and subjects of joy, deal with emotional and practical support and self-care. Providers: HF nurses Control Usual care, including traditional care in hospital and outpatient education and support	1. Patient perceived control (CAS) 2. Partners’ perceived control (CAS) 3. Patients’ Physical component score (SF-36) 4. Partners’ Physical component score (SF-36) 5. Patients’ Mental component score (SF-36) 6. Partners’ Mental component score (SF-36) 7. Patients’ Depression (Beck Depression Inventory) 8. Partners’ Depression (Beck Depression Inventory) 9. Patients’ self-care behaviour (EHFscBS) 10. Partners’ caregiver burden (CBS)	1. Statistically significant improvement in perceived control at 3 months in intervention group compared to control, but not at 12 months (*p* = 0.03). 2. No significant difference in either group. 3. No significant difference in either group. 4. No significant difference in either group. 5. No significant difference in either group. 6. No significant difference in either group. 7. No significant difference in either group. 8. No significant difference in either group. 9. No significant difference in either group. 10. No significant difference in either group.
**Aiken et al.** [35] **USA**	Phase III RCT, mixed population study Hospice of the Valley, Phoenix, Arizona (community based)	*N* = 100 (patients with CHF = 67) Mean Age of total population, no CHF subset analysis (SD) = 68 (14) Sex of total population, no CHF subset analysis: M 42%, F 58% All NYHA III or IV No information on LVEF	*N* = 90 (patients with CHF = 62) Mean Age of total population, no CHF subset analysis (SD) = 70 (13) Sex of total population, no CHF subset analysis: M 30%, F 70% All NYHA III or IV No information on LVEF	Intervention Intensive home-based case management. Program foci included disease and symptom management, patient self-management and knowledge of illness-related resources, preparation for end of life, physical and mental functioning and utilisation of medical services Providers: registered nurse case-managers, in coordination with primary care physician, community agencies, medical director, social worker & pastoral counsellor. Control Usual care provided by Managed Care Organisations (MCOs). Focus on medical and disease oriented approach, including medication and lab monitoring. Services delivered via telephone. Support services included disease and symptom education, nutrition and psychological counselling, transportation and co-ordination of medical services	1. Self-management of illness and knowledge of resources (self-developed questionnaire) 2. Preparation for end of life (self-developed questionnaire) 3. Participation in enjoyable activities (yes/no question) 4. Symptom control (Memorial symptom assessment scale) 5. Trajectories of physical and mental functioning (SF-36) 6. Medical utilisation (Emergency department visits and length of stay in hospital)	1. No CHF subset analysis. PhoenixCare participants had statistically significant sense of receiving sufficient information to handle illness emergency at 6 months (*p* < 0.05), sense of receiving education about community resources at 3 months (p < 0.05), information about who to talk to about medical problems at 6 months (p < 0.05) and better preparedness for daily experiences at 3 months (effect reversed by 6 months) (p < 0.05). 2. No CHF subset analysis. PhoenixCare participants had a statistically higher rate of completion of living will/advance directive after 3 months (p < 0.05). No significant difference at 6 months. 3. No significant difference in CHF participants. 4. No significant change in frequency of symptoms in CHF participants in PhoenixCare group. Significantly higher mean distress at 6 months in CHF participants in PhoenixCare compared to control (p < 0.05). 5. Stable physical scores in intervention group, while controls deteriorated at 9 months (p < 0.05). No difference in mental health scores. 6. No significant difference between groups.
**Bekel-man et al.** [36] **USA**	Multisite RCT Community based with outpatient consultations	*N* = 187 Mean Age (SD) = 68.3 (9.6) Sex: M 178 (95%), F 9 (5%) NYHA Class I 16 (8.9%) NYHA Class II 77 (42.8%) NYHA Class III 82 (45.6%) NYHA Class IV 5 (2.8%) LVEF: normal (78, 45.6%), 40–49% (34, 19.9%), 30–39% (46, 26.9%), < 30 (13, 7.6%)	*N* = 197 Mean Age (SD) = 67.9 (10.6) Sex: M 193 (98%), F 4 (2%) NYHA Class I 16 (8.5%) NYHA Class II 85 (45%) NYHA Class III 82 (43.4%) NYHA Class IV 6 (3.2%) LVEF: normal (84, 47.5%), 40–49% (34, 19.2%), 30–39% (32, 18.1%), < 30 (27, 15.3%)	Intervention 1) Multidisciplinary collaborative care: team met weekly to recommend changes based on telemonitoring data and PHQ-9 scores 2) Screening for and treatment of depression - those who screened positive received up to 11 sessions of behavioural activation and antidepressant management, a depression educational video and depression assessment & self-management education via telemonitoring 3) Daily telemonitoring of clinical observations, weight and self-reported symptoms & self-care program such as medication reminders, education about HF & dietary advice Providers: Nurse coordinator, primary care physician, cardiologist & psychiatrist Control Usual care provided by their regular health professionals, including telemonitoring if enrolled. Information sheets for self-care given and if screened positive for depression at baseline, primary care physicians were notified.	1. Patient-reported HF-specific Health Status (KCCQ) 2. Deaths 3. Depression (PHQ-9 score) 4. Hospitalisation rates	1. Statistically significant improvement in KCCQ overall summary scores in both groups after 1 year, with no significant difference between groups. 2. Fewer deaths in intervention group compared to control (*p* = 0.04). 3. Greater improvement in PHQ-9 score after 1 year in the intervention arm than usual arm if screened positive for depression (*p* = 0.01). 4. No significant difference in hospitalisations between intervention group and control group.
**Bekel-man et al.** [37] **USA**	Multisite RCT Outpatient consultations	*N* = 157 Mean Age (SD) = 64.5 (10.9) Sex: M 128 (81.5%), F 29 (18.5%) NYHA Class I 5 (3.2%) NYHA Class II 55 (35%) NYHA Class III 75 (47.8%) NYHA Class IV 20 (12.7%) LVEF: Normal (57/150, 38%), Mild (28/150, 18.7%), Moderate (30/150, 20%), Severe (35/150, 23.3%)	N = 157 Mean Age (SD) = 66.5 (11.8) Sex: M 119 (758%), F 38 (24.2%) NYHA Class I 12 (7.6%) NYHA Class II 46 (29.3%) NYHA Class III 75 (47.8%) NYHA Class IV 24 (15.3%) LVEF: Normal (64/149, 43%), Mild (18/149, 12.1%), Moderate (22/149, 14.8%), Severe (45/149, 30.2%)	Intervention 1) Registered nurse reviewed/assessed symptoms, applied motivational interviewing to promote changes in health behaviours, follow up telephone assessments 1-2x per month 2) Social worker provided structured telephone-based psychosocial intervention and address depression symptoms if present. Provided support to informal caregivers as needed 3) Weekly collaborative care team meetings Providers: Registered nurse, social worker, primary care clinician, cardiologist and palliative care (PC) physician Control Care at the discretion of their clinicians (could include care from cardiology, palliative care and mental health). Information sheet outlining self-care for HF. If screened positive for significant depression, patients and their clinicians were contacted.	1. Patient-reported HF-specific Health Status (KCCQ) 2. Depressive symptoms (PHQ-9) 3. Anxiety symptoms (GAD-7) 4. Overall symptom distress (GSDS score) 5. Pain (PEG score) 6. Fatigue (PROMIS score) 7. Dyspnoea (Dyspnoea score)	1. No statistically significant difference between groups. 2. Statistically significant improvement in depressive symptoms in intervention group at 6 months (*p* = 0.02) 3. Statistically significant difference in anxiety symptoms at 3 months in intervention group (*p* < 0.01), but no difference at 6 months. 4. No significant difference between groups. 5. No significant difference between groups. 6. Statistically significant improvement in fatigue scores in intervention group compared to control at 6 months (p = 0.02). 7. No significant difference between groups.
**Brann-strom et al.** [45] **Sweden**	RCT Community based with outpatient consultations	*N* = 36 Mean age (SD) = 81.9 (7.2) Sex: M 26 (72.2%), F 10 (27.8%) NYHA Class III 28 (77.8%) NYHA Class IV 8 (22.2%) LVEF: 40–49 13 (36.1%), 30–39 16 (44.4%), < 30 7 (19.4%)	N = 36 Mean age (SD) = 76.6 (10.2) Sex: M 25 (69.4%) F 11 (30.6%) NYHA Class III 23 (63.9%) NYHA Class IV 11 (30.6%) Unknown NYHA Class 2 (5.5%) LVEF: 40–49 12 (33.3%), 30–39 21 (58.3%), < 30 3 (8.3%)	Intervention PREFER intervention Multidisciplinary approach involving collaboration between specialists in palliative and heart failure care. Appointment with nurses for initial assessment followed by using a model for person-centred palliative care based on patient goals. Provision of total care including assessment of symptoms, quality of life, and risk, and registration into HF and palliative care registry. Subsequent access to home care visits by MDT. Providers: Specialist HF nurses, PC nurses, cardiologist, PC physician, physiotherapist & OT Control Usual care provided by GPs or doctors/nurses at HF clinic.	1. Health-related quality of life (EQ-5D) 2. Symptom burden (ESAS) 3. Disease-specific quality of life (KCCQ) 4. Functional classes (NYHA score) 5. Number of hospitalisations 6. Mortality 7. Resource utilisation (utilisation of visits, phone calls and prescriptions of clinicians	1. Age-adjusted health-related QoL significantly improved in intervention group compared to control (p = 0.02). 2. No significant difference between groups in overall score. Within group analysis of intervention group found significant improvement in nausea (p = 0.02). 3. No significant difference between groups. 4. More patients in the intervention group experienced an improvement in functional class (11 of 28; (*p* = 0.015). 5. Mean number of hospitalisations significantly lower in intervention group compared to control (*p* = 0.009) & mean number of days spent in hospital significantly lower in the intervention group (*p* = 0.011). 6. No significant difference between groups. 7. Significantly more nurse visits in intervention group compared to usual care (1075 vs 230;, *p* = 0.000). More phone calls and prescriptions by doctors in usual care group (108 vs 230; no *p* value given).
**Ekman et al.** [46] **Sweden**	Randomised control feasibility study Outpatient consultations	*N* = 79 No separate group breakdown for age or sex Mean age across groups (SD) = 80.3 (6.8) Sex (both groups): M 91 (58%), F 67 (42%) Mean NYHA class = 3.2 ± 0.5 Mean LVEF = 0.43 ± 0.18	N = 79 No separate group breakdown for age or sex Mean age across groups (SD) = 80.3 (6.8) Sex (both groups): M 91 (58%), F 67 (42%) Mean NYHA class = 3.2 ± 0.5 Mean LVEF = 0.38 ± 0.15	Intervention Nurse-monitored outpatient clinic run in co-operation with study doctors. Focus on patient’s understanding/ knowledge of condition and management, symptom control, psychosocial and lifestyle factors. Care was individually planned and goal-centered. Practical guidelines developed for nursing staff on symptoms, nutritional status, diet and ability of the patient to participate in self-care. Subsequent regular follow up telephone call contact. Providers: HF nurses and study doctors Control Usual care typically from GP and attendance at the Emergency Department.	1. Follow up time (months) 2. Functional status (NYHA class) 3. Hospital re-admissions 4. Mortality 5. Survival without readmission	1. No significant difference between groups. 2. Statistically significant improvement in NYHA class seen in both groups (Control *p* < 0.001, Intervention *p* < 0.05). 3. No significant difference between groups. 4. No significant difference between groups. 5. No significant difference between groups.
**Hopp et al.** [38] **USA**	Pilot RCT Three urban hospital sites	*N* = 43 Mean Age (SD) = 67.0 (11.0) Sex: M 26 (60.5%); F 17 (39.5%) No information on NYHA class Mean LVEF = 36.4 (16.7)	*N* = 42 Mean Age (SD) = 68.0 (13.0) Sex: M 18 (42.9%); F 24 (57.1%) No information on NYHA class Mean LVEF = 38.1 (16.8)	Intervention Palliative care consultation in hospital. Clinical interviews assessed for uncontrolled symptoms, goals of care, code status, ACP and desired post-treatment residential setting. Option to refer to other specialties (e.g. chaplaincy or social work) if indicated. Providers: Palliative care physician or advanced nurse practitioner Control No information	1. Election of “comfort care”: a outpatient hospice b inpatient hospice c DNR order during index or subsequent hospital admission d DNR order at home or nursing home 2. Mortality	1. 4 of 43 patients in intervention group had met primary end point, compared with 0 of 42 patients in control group; no statistically significant difference between groups. 2. No significant difference between groups.
**Liljeroos et al.** [47] **Sweden**	*Note: longer term follow up of Agren et al.* RCT Hospital and community based (outpatient clinic and in patients’ homes)	N = 71 dyads (patient & carer) Patients’ Mean Age (SD): 69 (13) Partners’ Mean Age (SD): 67 (12) Sex (patients): M 49 (69.1%), F 22 (30.9%) Sex (partners): M 22 (30.9%), F 49 (69.1%) NYHA Class II 25 (35%) NYHA Class III 39 (55%) NYHA Class IV 7 (10%) No information on LVEF	N = 84 dyads (patient & partner) Patients’ Mean Age (SD): 73 (10) Partners’ Mean Age (SD): 70 (10) Sex (patients): M 68 (80.9%), F 16 (19.1%) Sex (partners): M 16 (80.9%), F 68 (19.1%) NYHA Class II 25 (30%) NYHA Class III 43 (51%) NYHA Class IV 16 (19%) No information on LVEF	Intervention HF nurse-led face to face counselling, a computer based CD-ROM program and written teaching materials. 3 sessions in home or clinic: each included education on CHF, development of problem-solving skills in recognising factors contributing to psychological and emotional distress and focused on changing thoughts and behaviours and implementing strategies for self-care. Opportunity for dyads to receive information, raise questions, discuss difficulties and subjects of joy, deal with emotional and practical support and self-care. Providers: HF nurses Control Usual care, including traditional care in hospital and outpatient education and support	1. Time to first readmission/ death (days) 2. Number of readmissions 3. Number of days in hospital 4. Patients’ and partners’ physical component scores (SF-36) 5. Patients’ and partners’ mental component scores (SF-36) 6. Patients’ and partners’ physical functioning (unclear measurement tool) 7. Role limitations due to physical health problems 8. Bodily pain (unclear tool) 9. General health (unclear measurement tool) 1. Vitality (unclear tool) 2. Social functioning 3. Role limitations due to emotional problems 4. Depression (Beck depression inventory) 5. Perceived control (CAS)	1. No significant difference between groups. 2. No significant difference between groups. 3. No significant difference between groups. 4. No significant difference in patients’ PCS between groups. Significantly greater reduction in partners’ PCS in intervention group (*p* < 0.05). 5. No significant difference between groups. 6. No significant difference in patient’s functioning between groups. Significantly greater reduction in partners’ functioning in intervention group (p < 0.05). 7. No significant difference between groups. 8. No significant difference between groups. 1. No significant difference between groups. 2. No significant difference between groups. 3. No significant difference between groups. 4. No significant difference between groups. 5. No significant difference between groups. 6. No significant difference between groups.
**Mentz et al.** [39] **USA**	*Note: same population/ intervention as Rogers et al.* RCT Outpatient consultations	*N* = 75 Mean Age (SD) = 71.9 (12.4) Sex: M 42 (56%); F 33 (44%) NYHA Class III 54 (72%) NYHA Class IV 15 (20%) LVEF: Normal > 55% 21 (28%), 40–55% 14 (18.7%), 25–40% 17 (22.7%), < 25% 23 (30.7%)	N = 75 Mean Age (SD) = 69.8 (13.4) Sex: M 37 (49.3%); F 38 (50.7%) NYHA Class III 58 (77.3%) NYHA Class IV 5 (6.7%) LVEF: Normal > 55% 14 (18.7%), 40–55% 19 (25.3%), 25–40% 14 (18.7%), < 25% 28 (37.3%)	Intervention PAL-HF: interdisciplinary, multicomponent palliative care intervention. PC nurse practitioner coordinated patient’s care in collaboration with palliative care physician and the cardiology team. Pal-HF nurse participated in ongoing management in outpatient setting on hospital discharge. Focus on physical symptoms, psychosocial and spiritual concerns, ACP and shared goal-setting. Providers: PC nurse, PC physician, cardiology nurse Control Usual care. Cardiologist-directed team. Had access to inpatient PC if referred. Outpatient follow up with their general practitioners and HF cardiologist or nurse practitioner.	1. Total hospital admissions 2. Days alive and out of hospital 3. Admissions due to HF 4. Non cardiovascular-related admissions	1. No significant difference between groups. 2. No significant difference between groups. 3. No significant difference between groups. 4. No significant difference between groups.
**Ng et al.** [49] **China**	*Note: same population/ intervention as Wong et al. (2018)* RCT Community setting	N = 43 Mean Age (SD) = 78.3 (16.8) Sex: M 18 (43.9); F 25 (56.1%) NYHA class II = 6 (14.0%) NYHA class III = 31 (72.1%) NYHA class IV = 6 (14.0%) Mean LVEF = 39.0 (14.0)	*N* = 41 Mean Age (SD) = 78.4 (10.0) Sex: M 25 (61.0%); F 16 (39.0%) NYHA class II = 3 (7.3%) NYHA class III = 22 (53.7%) NYHA class IV = 16 (39.0%) Mean LVEF = 37.0 (17.0)	Intervention Post-hospital discharge home visits and telephone calls by palliative care nurse case managers and supported by trained volunteers (nursing students). Key components were physical and psychological symptom assessment and management, social support, spiritual and existential aspects of care, setting goals of care and discussion of treatment preferences and end-of-life issues. PC-nurse could refer to PC physician and other services as necessary. Providers: Palliative care nurse case managers, trained volunteers (nursing students), palliative care physician Control Pre-discharge PC referral consultation and scheduled outpatient PC clinic. Unstructured episodic home care service arranged if needed. 2 social calls from ‘assistant’ consisting of light conversation. Unclear who service delivered by.	1. Quality of life (McGill QoL Questionnaire-Hong Kong) 2. HF-specific quality of life (CHQ-C) 3. Symptom burden (ESAS) 4. Functional status (Palliative Performance Scale) 5. Patient satisfaction 6. Caregiver burden (ZBI)	1. Statistically significant improvement in QoL in intervention group compared to control (*p* = 0.016), with significant improvement in specific domains: physical (*p* = 0.011), psychological (*p* = 0.04), and existential (*p* = 0.027). 2. Statistically significant improvement in intervention group compared to control at 4 weeks (p = 0.01), but not at 12 weeks. Subset analysis of domains showed statistically significant difference in dyspnoea (p = 0.02), emotional function (*p* = 0.014) and mastery (*p* = 0.019) at 4 weeks, but not at 12 weeks. 3. No significant difference in overall score between groups. Subset analysis of domains showed statistically significant difference in depression (p = 0.01) and shortness of breath (*p* = 0.018) scores at 4 weeks in intervention group compared to control, but not at 12 weeks. 4. No significant difference between groups. 5. Statistically significant higher patient satisfaction score in intervention group compared to control group at 12 weeks (*p* = 0.001). 6. Statistically significant lower median score after 12 weeks in intervention group (*p* = 0.024).
**O’Don-nell et al.** [40] **USA**	RCT Hospital and community based	*N* = 26 Mean Age (SD) = 74.7 (11.2) Sex: M 14 (53.9%); F 12 (46.1%) No information on NYHA class Mean LVEF (SD) = 30 (14)	*N* = 24 Mean Age (SD) = 69.2 (10.2) Sex: M 15 (62.5%); F 9 (37.5%) No information on NYHA class Mean LVEF (SD) = 36 (17)	Intervention Initial palliative care SW consultation with assessment of prognostic understanding and preferences; a discussion of prognosis and its implications for decision making; an exploration of key topics including patient goals and priorities, fears and worries, sources of strength, perceived critical abilities, trade-offs between length and quality of life, and family awareness of preferences and prognosis; and summaryimpressions and recommendations. Education provided on importance of ACP, the role of health care proxy, and the need for other legal documents. Review by palliative care physician who facilitated further discussions and directed specific interventions. Telephone contact follow up from SW for next 6/12. Ongoing assessment of physical and psychological symptoms during course of follow up. Providers: Palliative care SW, palliative care physician Control Usual in-hospital and post-discharge treatment as directed by care team. Printed materials containing ACP information.	1. Documentation of advanced care preferences 2. Improvement in prognostic alignment (revision of patient expectations of prognosis in a direction consistent with those of the treating physician) 3. Physician-level ACP conversations (Presence of Medical Order for Life-sustaining treatment, advanced directive form or hospice referral) 4. Spiritual wellbeing (Functional Assessment of Chronic Illness Therapy-Spiritual Well-being) 5. QOL (abbreviated KCCQ) 6. Depression (Personal Health Questionnaire Depression Scale) 7. Anxiety (GAD-7)	1. Statistically significant increased proportion of patients had documented ACP in intervention group compared to control (p = 0.02). 2. Significantly more surviving patients in intervention group compared with control group had revised their baseline prognostic assessment in a direction consistent with the physician’s assessment (*p* < 0.001). 3. No significant difference between groups. 4. No significant difference between groups. 5. No significant difference between groups. 6. No significant difference between groups. 7. No significant difference between groups.
**O’Rior-dan et al.** [41] **USA**	Pilot RCT Hospital and outpatient based	*N* = 16 Mean Age (SD) = 71 (18) Sex: M 5 (31%); F 11 (69%) No separate group breakdown of NYHA class Total NYHA class: I 7 (23%), II 9 (30%), III 11 (37%), IV 3 (10%) No information on LVEF	N = 14 Mean Age (SD) = 59 (19) Sex: M 10 (62%); F 4 (28%) No separate group breakdown of NYHA class Total NYHA class: I 7 (23%), II 9 (30%), III 11 (37%), IV 3 (10%) No information on LVEF	Intervention SMS-HF: intensive palliative care consultations provided by an interdisciplinary palliative care team. Care included prescribing medications for symptoms, discussing ACP, completing documentation and providing psychosocial and spiritual support. First consultation occurred during hospitalisation. Providers: PC nurse practitioner, PC physician, social worker and chaplain Control Standard cardiology care	1. Heart-related quality of life (MLHFQ) 2. Functional status (FACIT-PAL) 3. Depression (HADS) 4. Anxiety (HADS) 5. Pain severity (BPI) 6. Dyspnoea (Borg scale) 7. Guideline-driven HF therapies 8. ACP documentation 9. Satisfaction with care	1. No significant difference between groups. 2. No significant difference between groups. 3. No significant difference between groups. 4. No significant difference between groups. 5. No significant difference between groups. 6. No significant difference between groups. 7. No significant difference between groups. 8. No significant difference between groups. 9. No significant difference between groups.
**Paes** [52] **UK**	Pilot RCT Outpatient consultations	N = 6 Mean Age (SD) = 73.2 (4.2) Sex: M 6 (100.0%); F 0 (0.0%) NYHA class III 3 (50.0%) NYHA class IV 3 (50%) No information on LVEF	N = 5 Mean Age (SD) = 78.0 (7.0) Sex: M 4 (80.0%); F 1 (20.0%) NYHA class III 3 (60.0%) NYHA class IV 2 (40%) No information on LVEF	Intervention Initial one hour outpatient consultation. Aim was to address: background, problematic physical and psychological symptoms, patient knowledge regarding their condition and prognosis, planning for the future, drug compliance, any issues raised by patient. Followed-up by monthly 30 min consultations for 5 months. Providers: Palliative care doctor Control Usual care provided by their consultant physician	1. Anxiety (HADS) 2. Depression (HADS) 3. Global health status and functional scale (EORTC QLQ-C30) 4. Symptom/problem scales (EORTC QLQ-C30) 5. Disease-specific QOL (KCCQ) 6. Disease-specific physical functioning (KCCQ) 7. Disease-specific symptom stability (KCCQ) 8. Social limitation (KCCQ) 9. Disease-specific functional status (KCCQ)	1. No significant difference between groups. 2. No significant difference between groups. 3. No significant difference between groups in any domains. 4. No significant difference between groups in any domains. 5. No significant difference between groups. 6. No significant difference between groups. 7. No significant difference between groups. 8. No significant difference between groups. 9. No significant difference between groups.
**Piam-jariyakul et al.** [42] **USA**	Pilot mixed methods study with randomised assignment of dyads to intervention or standard care Outpatient consultations	N = 10 dyads Patients’ Mean Age (SD) = 60.8 (14.5) Caregivers’ Mean Age (SD) = 65.1 (8.0) Sex (patients): M 6 (60%); F 4 (40%) Sex (caregivers): M 2 (20%); F 8 (80%) No information on NYHA class or LVEF	N = 10 dyads Patients’ Mean Age (SD) = 63.7 (13.1) Caregivers’ Mean Age (SD) = 57.3 (10.9) Sex (patients): M 6 (60%); F 4 (40%) Sex (caregivers): M 1 (10%); F 9 (90%) No information on NYHA class or LVEF	Intervention Post-hospital telephone coaching to caregivers on specific HF home care skills including symptom monitoring, medication adherence, sodium/fluid restrictions. Materials sent by mail: 1) Caregivers guide; 2) list of local support organisations; 3) national award winning book on a guide for caregivers in HF; 4) low sodium booklet; 5) pill organiser. 4 weekly coaching sessions included symptom control assessment, support for family (and SW referral if indicated), & content related to end-of-life care including advanced directive forms. Providers: HF nurse specialist Control Standard HF care provided by their physician and cardiologist including education materials routinely given to HF patients.	1. Patients’ HF-related hospitalisation frequency 2. Caregivers’ confidence in providing HF homecare (4-item Likert scale) 3. Caregivers’ preparedness in providing HF homecare (1-item Likert scale) 4. Perceived social support (1 item Likert scale) 5. Caregiver burden (17-item Likert scale) 6. Caregiver depression (CES-D)	1. Intervention group had a significant reduction in re-hospitalisations compared to control group (*p* = 0.03). 2. Significantly improved caregiver confidence in intervention group (*p* = 0.003). 3. No significant difference between groups. 4. Statistically significant greater perceived social support in intervention group compared to control (*p* = 0.01). 5. No significant difference between groups. 6. Statistically significant improved scores in intervention group compared to control (p = 0.01).
**Rogers et al.** [43] **USA**	RCT Outpatient consultations	N = 75 Mean Age (SD) = 71.9 (12.4) Sex: M 42 (56%); F 33 (44%) NYHA Class III 54 (72%) NYHA Class IV 15 (20%) LVEF: Normal > 55% 21 (28%), 40–55% 14 (18.7%), 25–40% 17 (22.7%), < 25% 23 (30.7%)	N = 75 Mean Age (SD) = 69.8 (13.4) Sex: M 37 (49.3%); F 38 (50.7%) NYHA Class III 58 (77.3%) NYHA Class IV 5 (6.7%) LVEF: Normal > 55% 14 (18.7%), 40–55% 19 (25.3%), 25–40% 14 (18.7%), < 25% 28 (37.3%)	Intervention PAL-HF: interdisciplinary, multicomponent palliative care intervention. Palliative care nurse practitioner coordinated patient’s care in collaboration with palliative care physician and the cardiology team. Pal-HF nurse participated in ongoing management in outpatient setting on hospital discharge. Focus on physical symptoms, psychosocial and spiritual concerns, ACP and shared goal-setting. Providers: PC nurse, PC physician, cardiology nurse Control Cardiologist-directed team. Had access to inpatient palliative care if referred. Outpatient follow up with their general practitioners and HF cardiologist or nurse practitioner.	1. HF-specific QOL (KCCQ) 2. General and palliative care-specific, health related QOL (FACIT-PAL) 3. Spiritual wellbeing (FACIT-Sp) 4. Depressive symptoms (HADS) 5. Anxiety symptoms (HADS) 6. Mortality 7. Hospitalisations	1. Statistically significant incremental improvement in scores in intervention group compared to control at 6 months (*p* = 0.030). 2. Statistically significant incremental improvement in scores in intervention group compared to control at 6 months (*p* = 0.035). 3. Statistically significant improvement in scores in intervention group (*p* = 0.027). 4. Statistically significant improvement in scores in intervention group (*p* = 0.020). 5. Statistically significant improvement in intervention group compared to control (*p* = 0.048) 6. No significant difference between groups. 7. No significant difference between groups.
**Sahlen et al.** [48] **Sweden**	*Note: substudy of Brannstrom et al.* RCT Community based with outpatient consultations	N = 36 Mean age (SD) = 81.9 (7.2) Sex: M 26 (72.2%), F 10 (27.8%) NYHA Class III 28 (77.8%) NYHA Class IV 8 (22.2%) LVEF: 40–49 13 (36.1%), 30–39 16 (44.4%), < 30 7 (19.4%)	N = 36 Mean age (SD) = 76.6 (10.2) Sex: M 25 (69.4%) F 11 (30.6%) NYHA Class III 23 (63.9%) NYHA Class IV 11 (30.6%) Unknown NYHA Class 2 (5.5%) LVEF: 40–49 12 (33.3%), 30–39 21 (58.3%), < 30 3 (8.3%)	Intervention PREFER intervention: MDT approach involving collaboration between specialists in palliative and HF care. Appointment with nurses for initial assessment followed by using a model for person-centred palliative care based on patient goals. Provision of total care including assessment of symptoms, QoL, and risk, and registration into HF and palliative care registry. Subsequent access to home care visits.Providers: Specialist HF nurses, PC nurses, cardiologist, PC physician, physiotherapist & OT Control Usual care provided by GPs or doctors/nurses at HF clinic.	1. Quality Adjusted Life Years (QALYs) 2. Costs of care (Euros €)	1. Intervention group had a slight improvement in QALYs and control group had a decline. These changes were small but significant (*p* = 0.026). Net gain of 0.25 QALYs with intervention. 2. The intervention reduced costs by €61,000 over intervention period (due to reduced hospital admissions).
**Wong et al.** [50] **China**	RCT Community setting	N = 43 Mean Age (SD) = 78.3 (16.8) Sex: M 18 (43.9); F 25 (56.1%) NYHA class II = 6 (14.0%) NYHA class III = 31 (72.1%) NYHA class IV = 6 (14.0%) Mean LVEF = 39.0 (14.0)	N = 41 Mean Age (SD) = 78.4 (10.0) Sex: M 25 (61.0%); F 16 (39.0%) NYHA class II = 3 (7.3%) NYHA class III = 22 (53.7%) NYHA class IV = 16 (39.0%) Mean LVEF = 37.0 (17.0)	Intervention Post-hospital discharge home visits and telephone calls by PC nurse case managers and supported by trained volunteers (nursing students). Key components were physical and psychological symptom assessment and management, social support, spiritual and existential aspects of care, setting goals of care and discussion of treatment preferences and end-of-life issues. PC-nurse could refer to PC physician and other services as necessary. Providers: PC nurse case managers, trained volunteers (nursing students), PC physician Control Pre-discharge PC referral consultation and scheduled outpatient PC clinic. Unstructured episodic home care service arranged if needed. 2 social calls from ‘assistant’ consisting of light conversation. Unclear who service delivered by.	1. Hospital readmissions 2. Symptom intensity (ESAS) 3. Functional status (Palliative Performance Scale) 4. Quality of life (MQOL-HK) 5. HF-specific quality of life (CHQ-C) 6. Satisfaction with care	1. Statistically significant reduction in readmissions in intervention group at 12 weeks (p 0.001). 2. Significantly higher clinical improvement in overall symptom intensity in intervention group compared with control group (*p* < 0.05). Domain analysis also showed significant difference in minimally clinically important difference of depression scores (p < 0.05) and dyspnoea scores (p < 0.05). 3. No significant difference between groups. 4. Statistically significant improvement in intervention group compared to control (p < 0.05). 5. Statistically significant improvement in intervention group compared to control (*p* < 0.01). 6. Statistically significant higher satisfaction with care in intervention group (*p* < 0.001).
**Wong et al.** [51] **China**	RCT Community setting	N = 43 Mean Age (SD) = 78.3 (16.8) Sex: M 18 (43.9); F 25 (56.1%) NYHA class II = 6 (14.0%) NYHA class III = 31 (72.1%) NYHA class IV = 6 (14.0%) Mean LVEF = 39.0 (14.0)	N = 41 Mean Age (SD) = 78.4 (10.0) Sex: M 25 (61.0%); F 16 (39.0%) NYHA class II = 3 (7.3%) NYHA class III = 22 (53.7%) NYHA class IV = 16 (39.0%) Mean LVEF = 37.0 (17.0)	Intervention Post-hospital discharge home visits and telephone calls by PC nurse case managers and supported by trained volunteers (nursing students). Key components were physical and psychological symptom assessment and management, social support, spiritual and existential aspects of care, setting goals of care and discussion of treatment preferences and end-of-life issues. PC-nurse could refer to PC physician and other services as necessary. Providers: PC nurse case managers, trained volunteers (nursing students), PC physician Control Pre-discharge PC referral consultation and scheduled outpatient PC clinic. Unstructured episodic home care service arranged if needed. 2 social calls from ‘assistant’ consisting of light conversation. Unclear who service delivered by.	1. Healthcare costs (Hong Kong dollar $) 2. Quality adjusted life years (QALYs)	1. Cost per case for intervention group was HK$7848/HK$10123 (28/84 days) compared with HK$15,783/HK$36,206 (28/84 days) in control group. Net incremental costs for intervention group was -HK$7935/−HK$26,084. 2. No statistically significant difference between groups.

### Participants

One thousand six hundred forty-two patients with CHF and 175 caregivers were included in all studies. Only three studies enrolled patient and caregiver dyads [42, 44, 47]. The mean age of all participants in included studies ranged from 59.0 to 81.9 years. The average age of included patients in the intervention group was 72.7 years and 72.0 years in the control group. The average age of included caregivers was 68.0 years in the intervention group and 70.0 in the control group. 17 studies provided figures for the sex of the 1513 patients with CHF, of which 1109 (73.3%) were male and 404 (26.7%) were female. Conversely, among 175 caregivers, most were female (*n* = 134; 76.6%). In addition, of the 1192 participants with NYHA classification data included in the 13 studies, 63 (5.3%) were Class I, 340 (28.5%) were Class II, 640 (53.7%) were Class III, and 149 (12.5%) were Class IV.

### Interventions

Table 2 outlines the palliative care components delivered in the intervention group in each study (at least two components required to meet inclusion criteria). The most frequently included components were optimising symptom control, which included meeting physical, psychological, social, and spiritual needs (*n* = 14), and advance care planning (*n* = 13). These were closely followed by organising multidisciplinary services (*n* = 11) and re-exploring goals of care (n = 11). No study provided a description of an intervention which delivered care after death, including bereavement support.

**Table 2:** Outline of palliative care components provided in included studies (*n* = 18)

	Optimising Evidence-Based Therapy	Breaking Bad News	Advance Care Planning	Education and Counselling on Self-Management	Organising Multidisciplinary Services	Identifying End-stage Heart Failure	Re-Exploring Goals of Care	Optimising Symptom Management	Care After Death
Agren et al. (2012) [44]	✓		✓	✓			✓		
Aiken et al. (2006) [35]	✓		✓	✓	✓			✓	
Bekelman et al. (2015) [36]	✓			✓	✓			✓	
Bekelman et al. (2018) [37]	✓				✓			✓	
Brannstrom et al. (2014) [45]				✓	✓		✓	✓	
Ekman et al. (1998) [46]				✓	✓				
Hopp et al. (2016) [38]			✓				✓	✓	
Liljeroos et al. (2015) [47]	✓		✓	✓			✓		
Mentz et al. (2018) [39]			✓		✓		✓	✓	
Ng et al. (2018) [49]			✓		✓	✓	✓	✓	
O’Donnell et al. (2018) [40]		✓	✓			✓	✓		
O’Riordan et al. (2019) [41]			✓					✓	
Paes (2005) [52]		✓	✓			✓		✓	
Piamjariyakul et al. (2015) [42]	✓		✓	✓				✓	
Rogers et al. (2017) [43]			✓		✓		✓	✓	
Sahlen et al. (2016) [48]				✓	✓		✓	✓	
Wong et al. (2016) [50]			✓		✓	✓	✓	✓	
Wong et al. (2018) [51]			✓		✓	✓	✓	✓	

There was a range of care providers involved across the intervention groups. These included interventions delivered by specialist cardiology teams (*n* = 4) [42, 44, 46, 47], by clinicians who had specialist palliative care expertise (*n* = 8) [35, 38, 40, 41, 49–52] and those interventions delivered using a collaborative approach with input from at least two of specialist cardiology, palliative care or primary care clinicians (*n* = 6) [36, 37, 39, 43, 45, 48].

### Comparators

One study did not provide any details of the comparator group used [38]. Of the remaining studies, the significant majority used a “usual care” group as control. This typically included care delivered either by their cardiology team or their primary care team, or both. Three studies included a comparator group which had access to a pre-discharge palliative care referral consultation and one scheduled outpatient palliative care clinic [49–51].

### Quality assessment

Only four studies demonstrated low risk of bias [36, 37, 49, 50], four were found to have some concerns regarding their risk of bias [38, 39, 42, 46] and the remaining ten studies were deemed to display overall high risk of bias [35, 40, 41, 43–45, 47, 48, 51, 52]. Nine studies were at high risk in the measurement of the outcome. Errors in measuring of participants’ outcome variables arise when the measured values do not equal the true or underlying values. These errors are often called measurement errors and risk of bias in this domain needs to consider the measurement tool used but also who the outcome assessor is, whether they are blinded to intervention allocation and whether the assessment of outcome is likely to be influenced by knowledge of intervention received [33]. Seven studies allowed patients to self-report outcome data, which led to a high risk of bias given they were un-blinded to the intervention and the measurements in question were at risk of subjectivity [40, 41, 43–45, 47, 52]. Other studies provided insufficient information to determine how and who collected data [48, 51].

Missing outcome measurements, for example due to dropouts, may lead to bias in the intervention effect estimate. Several studies had high rates of missing data which were unevenly distributed across intervention arms, thereby indicating a potential underlying reason for attrition dependent on allocation of treatment arm [35, 51, 52]. For example, Aiken et al. had a significant imbalance in missing data between intervention and control groups (44% of participants in the intervention group and 25% in the control group remained in the study). Moreover, the attrition analysis revealed that missing data was more likely in patients with poor physical outcomes (i.e., death, admission to hospice or skilled nursing facilities), implying a potentially inherent attrition bias between groups [35].

A summary of the risk of bias assessment for each included study is provided in Table 3, indicating risk of bias judgements across five core domains of the Cochrane RoB 2 Tool and an overall risk of bias judgement.

**Table 3:**
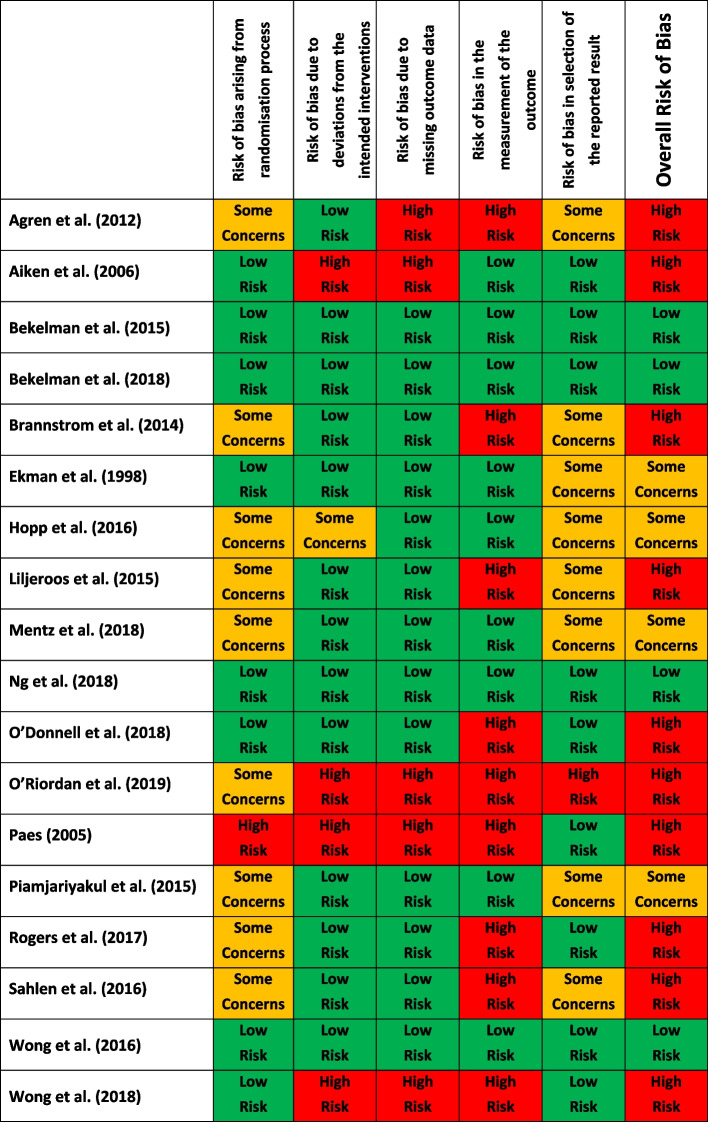
Summary of risk of bias assessment using Cochrane risk of bias tool for randomised trials (RoB2) [35–52]

### Outcomes

Across all studies, multiple outcome measures were collected using a heterogeneous range of measurement tools. Table 4 provides a summary of the main results for people with CHF and Table 5 for caregivers.

**Table 4:**
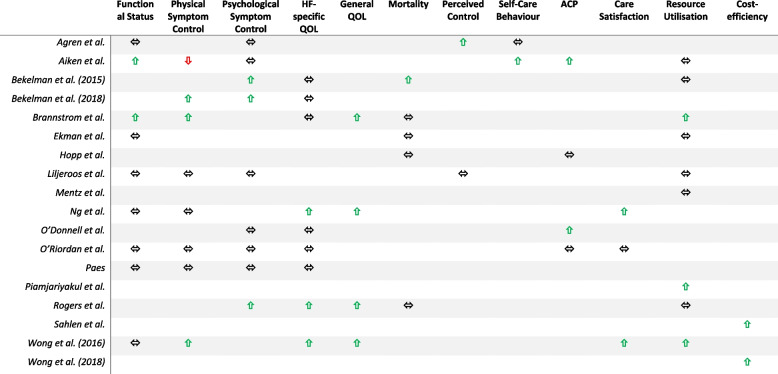
Summary of outcomes for people with chronic heart failure

**Table 5:**
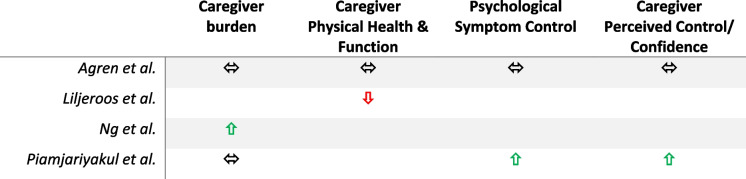
Summary of outcomes for caregivers of people with chronic heart failure

### Functional status

Nine studies reported on physical functioning of people with CHF [35, 41, 44–47, 49, 50, 52], with two demonstrating significant improvement in the intervention group compared to the control [35, 45]. Aiken et al. measured the physical component score of the SF-36 and found a statistically significant difference between arms whereby the intervention group remained functionally stable at 9 months, but the control group deteriorated (*p* < 0.05) [35]. Brannstrom et al. used the NYHA score and detected that more patients in the intervention group experienced an improvement in NYHA score compared to the control group (*p* = 0.015) [45]. All other studies assessing functional status found no significant difference between intervention and control groups, using various measures such as the Palliative Performance Status [49, 50], Functional Assessment of Chronic Illness Therapy - Palliative Care (FACIT-PAL) [41], and European Organisation for Research on Treatment of Cancer (EORTC QLQ-C30) [52].

### Physical and psychological symptom control

Eight studies assessed physical symptom burden [35, 37, 41, 45, 47, 49, 50, 52]. Overall, three out of eight studies showed some degree of statistically significantly improvement in physical symptom control in the intervention group compared to the control [37, 49, 50]. Wong et al. calculated the minimal clinically important difference of change in the Edmonton Symptom Assessment Scale (ESAS) scores and found that the intervention group experienced significantly higher clinical improvement in depression (45.9% vs 16.1%, *p* < 0.05), dyspnoea (62.2% vs 29.0%, p < 0.05) and total scores (73.0% vs 41.4%, *p* < 0.05) at 4 weeks [50]. Ng et al.’s study found no significant difference in overall ESAS scores between intervention and control groups over 12 weeks, however, a subset analysis did discover statistically significant improvement in dyspnoea after 4 weeks in the intervention arm (*p* = 0.018), but this was not maintained at 12 weeks [49]. Bekelman et al. measured the General Symptom Distress Scale (GSDS) and found no significant change in frequency of symptoms between intervention and control groups. However, fatigue, measured using the Patient-Reported Outcome Measurement Information System (PROMIS) score, showed a significant improvement in the intervention group at 6 months, with a mean difference between change scores of − 2.0 and a *p* value of 0.02 [37].

Nine studies measured psychological symptom control [35–37, 40, 41, 43, 44, 47, 52], of which three demonstrated improvements in the intervention group in comparison to the control [36, 37, 43]. Bekelman et al. reported a greater improvement in Patient Health Questionnaire (PHQ-9) depression scores in the intervention arm compared to the control if patients screened positive for depression on initial assessment (mean 2.1 points lower, *p* = 0.01) [36] and later in another study found an improvement in the intervention arm after 6 months in contrast to the control group (mean 1.2 points lower, *p* = 0.02) [37]. They also assessed anxiety symptoms using the Generalised Anxiety Disorder Assessment (GAD-7) tool and found whilst there was evidence of benefit in the intervention group after 3 months (*p* < 0.01), this was not maintained at 6 months. Rogers et al. recorded a statistically significant improvement in both depression and anxiety scores in the intervention compared with the control after 6 months (*p* = 0.02 and *p* = 0.048 respectively) using the Hospital Anxiety and Depression Scale (HADS) [43].

### Quality of life

Quality of life (QoL) was a commonly assessed outcome measure, with a total of nine studies reporting on it [36, 37, 40, 41, 43, 45, 49, 50, 52]. Many used a heart failure-specific measurement of QoL, such as the Kansas City Cardiomyopathy Questionnaire (KCCQ) [36, 37, 40, 45, 52], chronic heart failure questionnaire-Chinese (CHQ-C) [49, 50], or Minnesota Living with Heart Failure Questionnaire (MLHFQ) [41]. Three of the nine studies demonstrated significant improvement in heart failure-specific quality of life scores in the intervention arm [43, 49, 50]. Rogers et al. observed a statistically significant improvement in KCCQ scores in the intervention group compared to the control, with a mean score difference at 6 months of 9.49 (confidence interval 0.94–18.05, *p* = 0.030) [43]. Wong et al. (2016) showed that the mean score in the intervention group changed from 4.45 (standard error 0.14) at baseline to 5.26 (standard error 0.17) at 4 weeks, a statistically significant improvement when compared to the control group (*p* < 0.01) [50]. Similarly, there was a statistically significant improved CHQ-C score in the intervention arm compared to the control at 4 weeks demonstrated by Ng et al. (*p* = 0.01), however this was not maintained after 12 weeks [49].

Four studies also utilised a general QoL measure such as the McGill Quality of Life questionnaire – Hong Kong (MQOL-HK) [49, 50], EuroQoL-5D (EQ-5D) [45], or FACIT-PAL [43]. All four studies demonstrated a statistically significant improvement in the intervention arm [43, 45, 49, 50]. For example, Brannstrom et al. assessed health-related QoL utilising the EuroQol-5D (EQ-5D), a patient-rated five-dimension instrument, and found that between group analysis of the age-adjusted delta value from baseline to 6 months was significantly better in intervention group (*p* = 0.02) [45]. Rogers et al.’s study used the FACIT-PAL tool, and demonstrated a statistically significant mean score difference between intervention and control groups at 6 months of 11.77 (confidence interval 0.84–22.71, *p* = 0.035) [43].

### Perceived control and self-care behaviour

Two studies used the Control Attitude Scale (CAS) to measure level of perceived control [44, 47]. Agren et al. did find a statistically significant improvement in perceived control using CAS at 3 months in the intervention group (p = 0.03), but this was not maintained at 12 months [44]. Liljeroos were unable to detect any statistically significant difference between arms [47]. Moreover, Agren et al. found no significant difference in self-care behaviours between groups [44]. Aiken et al. also aimed to measure self-management outcomes using a self-developed questionnaire. Unfortunately, no subset analysis for the CHF population was performed, however, for all participants in the intervention group, they found there was a statistically significant improved sense of receiving sufficient information to handle illness emergency at 6 months, sense of receiving education about community resources at 3 months, information about who to talk to about medical problem at 6 months and better preparedness for daily experiences at 3 months (although this effect was reversed at 6 months) with a *p* value of < 0.05 [35].

### Satisfaction with care

Both Wong et al.’ and Ng et al.’s studies found statistically significantly higher levels of patient satisfaction with care in the intervention groups compared to the control groups at 4 weeks and 12 weeks respectively (*p* = 0.001 and *p* < 0.001) [49, 50]. O’Riordan et al.’ study found no difference between groups [41].

### Mortality

Five studies reported on mortality of their patient participants as part of their outcome measures [36, 38, 43, 45, 46]. Bekelman et al. found there were statistically significantly fewer deaths in the intervention group compared to the control group over the 12-month study period (*p* = 0.04) [36]. However, no significant difference was evidenced between groups in the remaining four studies [38, 43, 45, 46].

### Advance care planning

Four studies collected data on the presence of advance care planning (ACP) [35, 38, 40, 41]. Aiken et al. found a statistically higher rate of completion of either living wills or advance directives in the intervention group at 3 months compared to the control group (19% increase vs 4% increase, *p* < 0.05), but there was no significant difference at 6 months [35]. Likewise, O’Donnell et al. demonstrated that 17/26 patients (65%) in the intervention group and 8/24 (33%) patients in control group had documentation by their usual practitioner of advance care preference after 6 months, which was a statistically significant difference (*p* = 0.02). However, there was no significant difference between arms in the presence of orders for life-sustaining treatment, formal advanced directives or hospice referrals [40]. Moreover, both Hopp et al.’ and O’Riordan et al.’ studies found no significant difference between intervention and control groups in the presence of documented ACP (e.g., the presence of hospice referrals or ‘Do Not Attempt Resuscitation’ orders) [38, 41].

### Resource use and cost effectiveness

Nine studies provided outcome data on medical utilisation through a variety of measures [35, 36, 39, 42, 43, 45–47, 50]. Brannstrom et al. found that over a 6-month period, the mean number of hospitalisations were significantly lower in the intervention group compared to the control (0.42 ± 0.60 vs 1.47 ± 1.81, *p* = 0.009) and the mean number of days spent in hospital was significantly lower in the intervention group (2.9 ± 8.3 vs 8.5 ± 12.4, *p* = 0.011). Moreover, they demonstrated significantly higher rates of nurse home visits in the intervention group (1075 vs 230, *p* = 0.000) [45]. Piamjariyakul et al. found a significant reduction in re-hospitalisations in the intervention group compared to the control group (*p* = 0.03) [42]. Likewise, Wong et al. demonstrated significantly reduced hospital re-admission rates in the intervention group compared to the control over a 12-week period (*p* = 0.001) [50]. The remaining six studies failed to show any statistically significant difference in rates of hospitalisations or medical utilisation.

Two studies provided outcome data on economic costs, and both found the intervention group to be statistically significantly more cost effective than the control group, largely due to reduced rates of hospital admissions [48, 51]. Sahlen et al.’s study found the costs for staffing were significantly higher in the intervention group, however, this was offset by the significantly lower costs related to reduced hospital and emergency care. This led to an overall reduced cost of the intervention of €61,000 over the 6-month study period [48]. Wong et al. (2018) found that over the total 84-day study period, the total costs per case was HK$10,123 for the intervention group versus HK$36,206 in the control group. They calculated that the chance of the intervention group being cost-effective at 84 days was 100% [51].

With regards to quality-adjusted life years (QALYs), Sahlen et al. found that the intervention group had a slight improvement in QALYs and the control group had a decline. These changes were significant (*p* = 0.026) and there was a net gain of 0.25 QALYs associated with the intervention [48]. Wong et al. (2018) found no statistically significant difference between QALY gain in the intervention group compared to the control group [51].

### Caregiver outcomes

Only four studies provided outcome data related to caregivers [42, 44, 47, 49]. Of these, one study showed statistically significant improved caregiver burden related to the intervention group [49]. Piamjariyakul et al. did demonstrate improved levels of confidence in providing homecare (*p* = 0.003) and of perceived social support (*p* = 0.01) after 6 months, as well as statistically significantly improved scores in caregiver depression in the intervention arm compared to the control after 6 months (p = 0.01) [42]. Other studies either showed no significant difference in caregiver outcomes [44] or a statistically significant reduction in caregiver physical component scores in the intervention group after 24 months (*p* < 0.05), correlating with deteriorating physical health [47].

## Discussion

To our best knowledge, this is the first systematic review aimed at identifying and critically appraising the available evidence for the effectiveness and cost-effectiveness of palliative care interventions in both people with CHF and their caregivers. There are significant variations across the 18 included studies in terms of settings, interventions used, and outcomes measured. The most commonly assessed outcome measures were functional status (*n* = 9), psychological symptoms (*n* = 9), disease-specific quality of life (n = 9), and physical symptom control (*n* = 8). Moreover, the methodological quality of these studies was mixed, with only four having an overall low risk of bias and the remaining studies either demonstrating high risk of bias (*n* = 10) or showing some concerns (*n* = 4). Small sample sizes and poor retention were common. Only six studies were powered to detect effects of the intervention [37, 42, 45, 47, 49, 50].

The symptom burden in CHF is a significant issue. One study demonstrated that more than half of patients experienced moderate to severe breathlessness, fatigue, coughing, muscle weakness, sleeplessness, and low mood [53]. There is also evidence that symptom burden, quality of life and emotional wellbeing scores in CHF patients are no different to those seen in a cancer population [54]. Moreover, a US study found that patients with advanced heart failure had a statistically significant higher number of physical symptoms, higher depression scores and lower spiritual well-being than patients with advanced lung or pancreatic cancer [55]. Early palliative care led to significant improvements in both quality of life and mood among patients with metastatic non–small-cell lung cancer. As compared with patients receiving standard care, patients receiving early palliative care had less aggressive care at the end of life but longer survival [20].

In this review, whilst comparison of individual people with CHF and caregiver outcomes across studies does indicate inconsistency in results, it has highlighted the presence of a degree of evidence for the benefit of palliative care interventions for various outcomes for both people with CHF and their caregivers. The domains with the greatest evidence base include general and heart failure-specific quality of life, psychological symptom control, satisfaction with care, physical symptom control, cost-effectiveness, hospitalisation rates and medical utilisation, and caregiver burden. Moreover, it was rare for the palliative care interventions to confer negative effects. In addition, there is evidence highlighting that palliative care interventions can be provided across a range of settings and by multiple different specialities, most frequently through a collaborative approach. Indeed, the review has shown evidence that palliative care interventions can be successfully delivered by clinicians in specialties outside of palliative care.

For those studies that reported on NYHA staging, most people with CHF had stage III disease (54%). This is four times as many as there were with stage IV disease (13%) and suggests that those with the most severe disease are less likely to be recruited for research. This is perhaps a concern if one correlates advanced disease as a marker for increased need. Consequently, it may mean that the palliative care interventions of included studies were not reaching those for whom they will have maximum benefit and therefore potentially reduce intervention effect. However, this argument potentially places too much emphasis on the correlation between NYHA stage and palliative care need, given it has been shown that NYHA status fails to capture the symptom volatility of CHF [56]. In addition, a number of studies included control groups which had some element of palliative care provision, creating the possibility of contamination between intervention and control arms [37, 39, 43, 44, 47, 49–51]. Therefore, there is the risk that treatment effect of the palliative care interventions may have been diminished and the results of this review should be interpreted and implemented with caution.

Numerous outcome measures were used across the included studies in this review. Arguably, the validated tools assessing quality of life, physical and psychological symptoms are highly relevant. However, in the domain of assessing efficacy in supporting advanced care planning, it is debatable whether meaningful measures were used. For example, Aiken et al. measured the completion rate of living wills or advance directives [35]. When considered by a Delphi panel, formal advance directive documentation was only ranked the tenth most important outcome construct in defining successful ACP; with *“care consistent with patient goals”* ranking first [57]. Moreover, within other domains, there is perhaps a need for unifying consensus on the measurement tools which best evaluate the effectiveness and efficacy of palliative care interventions for both patients and their caregivers. For example, there has been a growing evidence base for the utility of patient-reported outcome measures in palliative care populations, such as the Integrated Palliative care Outcome Scale (IPOS) [58]. With respect to caregiver outcome measures, research into this area in a palliative care population is rather limited, and historically, little formal psychometric testing has been performed [59].

This review has highlighted several key areas where there is limited evidence which warrants further investigation. In terms of the patient population, older female patients are under-represented in studies. This may be no coincidence given women tend to develop heart failure at an older age [60]. However, the concern is that we are therefore potentially failing to quantify the extent to which palliative care interventions are effective in a large cohort of patients. Moreover, this is particularly troublesome given evidence shows that women with heart failure are often more symptomatic than men and therefore may have greater palliative care needs [61].

Another group under-represented in the evidence-base demonstrated by this review is caregivers. In total, the results of only 175 caregivers were eligible for inclusion in the review. Moreover, the majority of these studies had a high risk of bias. Therefore, the potential to draw conclusions on the effectiveness of palliative care interventions on outcomes in caregivers of patients with heart failure is lessened. Considering evidence indicates the high degree of caregiver needs in those caring for patients with heart failure, this is a highly significant gap for future research [62].

It is also apparent that no included studies contained a palliative care intervention that involved any aspect of care related to care after death, including bereavement services, and no study measured any outcomes pertaining to quality of death or quality of care after death. Arguably this is important given care in the final days of life and bereavement support are seen as key principles of palliative care. Therefore, it is impossible to be confident, based on this review, that there is evidence for the benefit of palliative care interventions on quality of death in patients with heart failure or support for their caregivers after death.

### Limitations

Non-English language studies were excluded due to lack of resources for translation services, which may have impacted on the results of the review. A definition of a palliative care intervention was used that encompassed the views of international guidelines and did not limit itself to requiring clinicians with palliative care as their core business. This meant the definition was relevant to the heart failure population and took into account the holistic nature of palliative care. However, it does lead to challenges in interpretation of results on the basis that the interventions included are highly diverse and therefore difficult to synthesise. Moreover, when there are so many components that constitute palliative care, it makes it challenging to quantify which component (or combination of components) is the effective element of the intervention.

In addition, only two studies evaluated the cost effectiveness, which was measured by the healthcare costs including medical costs attributable to emergency department attendance and length of stay in hospital [48, 51]. Calculation of the actual cost of the palliative care interventions such as professionals’ time spent in activities was based on an estimation of time spent for each patient by each staff category, multiplied by the average salaries of the professionals providing them. Therefore, the cost data in both studies may not be accurate to provide robust evidence on the cost effectiveness of palliative care interventions.

## Conclusions

This review has demonstrated that palliative care interventions have been found to benefit patients with chronic heart failure and their caregivers in a number of important domains, including improving general and heart failure-specific quality of life, psychological symptom control, satisfaction with care, physical symptom control, hospitalisation rates and medical utilisation, and caregiver burden. Therefore, in keeping with national and international guidelines, it is appropriate to recommend the role of palliative care interventions in this patient and caregiver group. Moreover, the review has provided evidence that palliative care interventions can be cost-effective when compared to usual care, primarily through reduction of acute hospital admissions. However, the methodological quality of the studies in this field to date are mixed and this has led to inconclusive results.

Furthermore, the question of who is best placed to deliver these palliative care interventions remains unclear. However, this review has provided evidence that they can be effectively delivered without necessarily requiring direct input from specialist palliative care clinicians. In order for generalists and cardiology teams to be able to deliver palliative care interventions, it is essential that they have adequate training and education to identify unmet palliative care needs, and develop and provide any innovative interventions to address these needs. Given most studies made use of a combination of professionals working in collaboration, a collaborative model of care in the heart failure population may be a way forward. This could allow for sharing of knowledge and expertise, co-ordination of care, improved inter-disciplinary working and the potential for up-skilling of non-specialists through shared learning.

There are significant evidence gaps that require further research, particularly in the areas of at what point palliative care interventions provide greatest benefit and the extent to which these interventions should be provided by specialists in palliative care versus non-specialists, alongside a collaborative approach. The evidence base for when palliative care interventions should take place in the heart failure population remains uncertain and this review has failed to clarify this. In other disease groups such as cancer, there is evidence for the effectiveness of early access to palliative care interventions in the disease trajectory. However, perhaps the question of time since diagnosis is less important than that of need*.*

## Supplementary Information

**Additional file 1.**

## Data Availability

All data generated or analysed during this study are included in this published article.

## Abbreviations

ACP: Advance Care Planning
BPI: Brief Pain Inventory
CAS: Control Attitude Scale
CBAs: Controlled before-and-after studies
CBS: Caregiver Burden Scale
CENTRAL: Cochrane Central Register of Controlled Trials
CES-D: Centre for Epidemiologic Studies Depression Scale
CHF: Chronic heart failure
CHQ-C: Chronic heart failure questionnaire-Chinese
EHFscBS: European Heart Failure Self-Care Behaviour Scale
EORTC QLQ-C30: European Organisation for Research on Treatment of Cancer
ESAS: Edmonton Symptom Assessment Scale
EuroQoL-5D: EQ-5D
FACIT-PAL: Functional Assessment of Chronic Illness Therapy – Palliative Care
FACIT-sp: FACIT Spiritual wellbeing scale
GAD-7: Generalised Anxiety Disorder Assessment
GSDS: General Symptom Distress Scale
HADS: Hospital Anxiety and Depression Scale
HMIC: Health management Information Consortium
ITS: Interrupted time series studies
KCCQ: Kansas City Cardiomyopathy Questionnaire
LVEF: Left Ventricular Ejection Fraction
MLHFQ: Minnesota Living with Hearth Failure Questionnaire
MQOL-HK: McGill Quality of Life questionnaire-Hong Kong
NYHA: New York Heart Association
PC: Palliative Care
PEG score: Pain, Enjoyment, General Activity score
PHQ-9: Patient Health Questionnaire
PRISMA: Preferred Reporting Items for Systematic reviews and Meta-Analyses
PROMIS: Patient-Reported Outcome Measurement Information System
QALYs: Quality-adjusted life years
QoL: Quality of Life
RCT: Randomised controlled trial
RoB 2: Revised Cochrane risk-of-bias tool for randomized trials
SD: Standard Deviation
SF-36: Short form 36
SW: Social worker
WHO: World Health Organization
ZBI: Zarit Burden Interview
