# Supplementary material for: Effectiveness and cost effectiveness of palliative care interventions in people with chronic heart failure and their caregivers: a systematic review
Source: BMC Palliat Care. 2022 Nov 23;21:205. doi: 10.1186/s12904-022-01092-2 (PMC9685889; doi:10.1186/s12904-022-01092-2)
Supplement: Supplementary file 1 — Additional file 1. [file 12904_2022_1092_MOESM1_ESM.docx]

Appendix 1: Full list of search terms

Medline

1. exp Heart Failure/

2. heart failure.mp.

3. chronic heart failure.mp.

4. congestive cardiac failure.mp.

5. congestive heart failure.mp.

6. cardiac failure.mp.

7. (advanced adj6 heart failure).mp.

8. myocard* failure.mp.

9. ventric* failure.mp.

10. dilated cardiomyopathy.mp.

11. cardiac insufficiency.mp.

12. myocard* insufficiency.mp.

13. ventric* insufficiency.mp.

14. 1 or 2 or 3 or 4 or 5 or 6 or 7 or 8 or 9 or 10 or 11 or 12 or 13

15. exp Palliative Care/

16. exp Hospice Care/

17. exp Terminal Care/

18. palliat*.mp.

19. hospice*.mp.

20. supportive care.mp.

21. "end of life".mp.

22. exp Advance Care Planning/

23. (bereavement adj2 support).mp.

24. caregiver support.mp.

25. 15 or 16 or 17 or 18 or 19 or 20 or 21 or 22 or 23 or 24

26. exp Randomized Controlled Trials as Topic/

27. exp Controlled Clinical Trial/

28. random*.mp.

29. rct.mp.

30. trial.mp.

31. 26 or 27 or 28 or 29 or 30

32. 14 and 25 and 31

Embase

1. exp Heart Failure/

2. heart failure.mp.

3. chronic heart failure.mp.

4. congestive cardiac failure.mp.

5. congestive heart failure.mp.

6. cardiac failure.mp.

7. (advanced adj6 heart failure).mp.

8. myocard* failure.mp.

9. ventric* failure.mp.

10. dilated cardiomyopathy.mp.

11. cardiac insufficiency.mp.

12. myocard* insufficiency.mp.

13. ventric* insufficiency.mp.

14. 1 or 2 or 3 or 4 or 5 or 6 or 7 or 8 or 9 or 10 or 11 or 12 or 13

15. exp Palliative Care/

16. exp Hospice Care/

17. exp Terminal Care/

18. palliat*.mp.

19. hospice*.mp.

20. supportive care.mp.

21. "end of life".mp.

22. exp Advance Care Planning/

23. (bereavement adj2 support).mp.

24. caregiver support.mp.

25. 15 or 16 or 17 or 18 or 19 or 20 or 21 or 22 or 23 or 24

26. exp Randomized Controlled Trials as Topic/

27. exp Controlled Clinical Trial/

28. random*.mp.

29. rct.mp.

30. trial.mp.

31. 26 or 27 or 28 or 29 or 30

32. 14 and 25 and 31

PsychINFO

1. heart failure.mp.

2. chronic heart failure.mp.

3. congestive cardiac failure.mp.

4. congestive heart failure.mp.

5. cardiac failure.mp.

6. (advanced adj6 heart failure).mp.

7. myocard* failure.mp

8. ventric* failure.mp

9. dilated cardiomypathy.mp.

10. cardiac insufficiency.mp

11. myocard* insufficiency.mp

12. ventric* insufficiency

13. 1 or 2 or 3 or 4 or 5 or 6 or 7 or 8 or 9 or 10 or 11 or 12

14. exp palliative care/

15. exp hospice/

16. exp terminally ill patients/

17. palliat*.mp

18. hospice*.mp

19. supportive care.mp

20. end of life care.mp

21. advance care planning.mp

22. (bereavement adj2 support).mp

23. caregiver support.mp

24. 14 or 15 or 16 or 17 or 18 or 19 or 20 or 21 or 22 or 23

25. exp Intervention/

26. exp Clinical Trials/

27. random*.mp

28. randomized controlled trial.mp

29. rct.mp

30. trial.mp.

31. 25 or 26 or 27 or 28 or 29 or 30

32. 13 and 24 and 31

CINAHL

S1 (MH “Heart Failure”)

S2 “cardiac failure”

S3 “myocard* failure”

S4 “ventric* failure”

S5 (MH “Cardiomyopathy, Dilated”)

S6 “cardiac insufficiency”

S7 “myocard* insufficiency”

S8 “ventric* insufficiency”

S9 S1 or S2 or S3 or S4 or S5 or S6 or S7 or S8

S10 (MH "Palliative Care") OR (MH "Hospice and Palliative Nursing")

S11 “hospice*”

S12 “palliat*”

S13 “end of life”

S14 “supportive care”

S15 (MH “Advance Care Planning”)

S16 “bereavement support”

S17 S10 or S11 or S12 or S13 or S14 or S15 or S16

S18 (MH "Randomized Controlled Trials")

S19 (MH "Clinical Trials")

S20 "randomised"

S21 "randomized"

S22 “RCT”

S23 S18 or S19 or S20 or S21 or S22

S24 S9 and S17 and S23

The Cochrane Central Register of Controlled Trials (CENTRAL)

#1 MeSH descriptor: [Heart Failure]

#2 cardiac failure

#3 chronic heart failure

#4 congestive cardiac failure

#5 #1 or #2 or #3 or #4

#6 MeSH descriptor: [Palliative Care]

#7 palliative

#8 hospice*

#9 terminal*

#10 bereavement

#11 caregiver support

#12 MeSH descriptor: [Terminal Care]

#13 #6 or #7 or #8 or #9 #10 or #11 or #12

#14 #5 and #13

Health Management Information Consortium (HMIC)

1. exp Heart Failure/

2. heart failure.mp.

3. chronic heart failure.mp.

4. congestive cardiac failure.mp.

5. congestive heart failure.mp.

6. cardiac failure.mp.

7. (advanced adj6 heart failure).mp.

8. myocard* failure.mp.

9. ventric* failure.mp.

10. dilated cardiomyopathy.mp.

11. cardiac insufficiency.mp.

12. myocard* insufficiency.mp.

13. ventric* insufficiency.mp.

14. 1 or 2 or 3 or 4 or 5 or 6 or 7 or 8 or 9 or 10 or 11 or 12 or 13

15. exp Palliative Care/

16. exp Hospice Care/

17. exp Terminal Care/

18. palliat*.mp.

19. hospice*.mp.

20. supportive care.mp.

21. "end of life".mp.

22. exp Advance Care Planning/

23. (bereavement adj2 support).mp.

24. caregiver support.mp.

25. 15 or 16 or 17 or 18 or 19 or 20 or 21 or 22 or 23 or 24

26. exp Randomized Controlled Trials as Topic/

27. exp Controlled Clinical Trial/

28. random*.mp.

29. rct.mp.

30. trial.mp.

31. 26 or 27 or 28 or 29 or 30

32. 14 and 25 and 31
